# Establishment of Patient-Derived Organoids from Hepatocellular Carcinoma: Preliminary Data on Yield, Histopathological Concordance, and Methodological Challenges

**DOI:** 10.3390/cells15020125

**Published:** 2026-01-10

**Authors:** Oriana Lo Re, Christian Corti, Lucia Cerrito, Eleonora Cesari, Elisabetta Creta, Flavio De Maio, Alessia Di Prima, Vincenzo Facciuto, Clelia Ferraro, Eleonora Huqi, Rosa Liotta, Margot Lo Pinto, Duilio Pagano, Riccardo Perriera, Valentina Petito, Giulia Santarelli, Francesco Santopaolo, Leonardo Stella, Floriana Tortomasi, Claudio Sette, Salvatore Gruttadauria, Felice Giuliante, Giovanni Zito, Francesca Romana Ponziani

**Affiliations:** 1 Istituto Mediterraneo per i Trapianti e Terapie ad Alta Specializzazione ISMETT IRCCS, 90127 Palermo, Italy; olore@ismett.edu (O.L.R.); adiprima@ismett.edu (A.D.P.); cleliaferr93@gmail.com (C.F.); rosa.liotta@ismett.edu (R.L.); mlopinto@ismett.edu (M.L.P.); dpagano@ismett.edu (D.P.); riccardo.perriera@ismett.edu (R.P.); ftortomasi@ismett.edu (F.T.); sgruttadauria@ismett.edu (S.G.); gzito@ismett.edu (G.Z.); 2 GSTep Organoids Research Core Facility, Fondazione Policlinico Universitario Agostino Gemelli IRCCS, 00168 Rome, Italy; christian.corti@guest.policlinicogemelli.it (C.C.); eleonora.cesari@policlinicogemelli.it (E.C.); claudio.sette@unicatt.it (C.S.); 3 Liver Unit, CeMAD Centro Malattie dell’Apparato Digerente, Medicina Interna e Gastroenterologia, Fondazione Policlinico Universitario Agostino Gemelli IRCCS, 00168 Rome, Italy; lucia.cerrito@policlinicogemelli.it (L.C.); elisabetta.creta@policlinicogemelli.it (E.C.); vincenzo.facciuto@guest.policlinicogemelli.it (V.F.); francesco.santopaolo@policlinicogemelli.it (F.S.); leonardo.dr.stella@gmail.com (L.S.); 4 Department of Laboratory and Hematological Sciences, Fondazione Policlinico Universitario Agostino Gemelli IRCCS, 00168 Rome, Italy; flavio.demaio@policlinicogemelli.it (F.D.M.); giulia.santarelli@policlinicogemelli.it (G.S.); 5 Microbiota Analysis & Microbial WGS Research Core Facility, GSTeP, Fondazione Policlinico Universitario Agostino Gemelli IRCCS, 00168 Rome, Italy; 6 CeMAD Translational Research Laboratories, Digestive Disease Center (CeMAD), Department of Medical and Surgical Sciences, Fondazione Policlinico Universitario Agostino Gemelli IRCCS, 00168 Rome, Italy; eleonora.huqi@guest.policlinicogemelli.it (E.H.); valentina.petito@policlinicogemelli.it (V.P.); 7 University of Pittsburgh Medical Center UPMC Italy, 90127 Palermo, Italy; 8 Department of Life Science, Link Campus University, 00165 Rome, Italy; 9 Department of Neuroscience, Section of Human Anatomy, Catholic University of the Sacred Heart, 00168 Rome, Italy; 10 Department of Surgery and Medical and Surgical Specialties, University of Catania, 95124 Catania, Italy; 11 Hepatobiliary Surgery Unit, Fondazione Policlinico Universitario Agostino Gemelli IRCCS, 00168 Rome, Italy; felice.giuliante@unicatt.it; 12 Dipartimento di Medicina e Chirurgia Traslazionale, Università Cattolica del Sacro Cuore, 00168 Rome, Italy

**Keywords:** hepatocellular carcinoma, organoids, liver tumor, cirrhosis, translational research

## Abstract

Patient-derived organoids (PDOs) have emerged as promising preclinical models for studying tumor biology and testing therapeutic strategies in oncology. These three-dimensional culture systems retain key histological, genetic, and functional characteristics of the original tumors, offering a unique opportunity to advance personalized medicine approaches in liver cancer. In this study, we present the methodological framework and preliminary findings of a prospective study aimed at generating and characterizing PDOs from patients with hepatocellular carcinoma (HCC) undergoing surgical resection. Tumor specimens were processed using an optimized protocol for organoid derivation, expansion, and cryopreservation. We evaluated the success rate of organoid establishment and the histo-molecular fidelity to the parental tumor. These early results demonstrate promising engraftment efficiency and maintenance of tumor-specific markers across passages. Our work highlights the potential of PDOs as a reliable and scalable platform for translational research in HCC, setting the stage for future applications in drug screening and biomarker discovery.

## Simple Summary

Patient-derived organoids (PDOs) are emerging as powerful preclinical tools for modeling hepatocellular carcinoma (HCC). However, current data on their establishment efficiency, histopathological fidelity, and immunophenotypic correspondence remain limited and inconsistent. In this study, we present preliminary data from a PDO platform derived from HCC specimens, with an in-depth focus on methodological aspects, success rates, and comparison with the original tumor. Our findings aim to support reproducibility and offer a potential benchmark for future research in the field of organoid-based precision oncology.

## 1. Introduction

Hepatocellular carcinoma (HCC) represents a major global health challenge, ranking as the sixth most common malignancy and the third leading cause of cancer-related mortality worldwide [1].

Despite improvements in early detection and therapeutic approaches, the prognosis of HCC remains poor, particularly in patients diagnosed at advanced stages, whose median survival ranges from 12 to 18 months [2]. HCC most commonly develops in the setting of chronic liver disease, primarily cirrhosis caused by viral hepatitis, alcohol-related liver disease, or the increasingly prevalent metabolic-dysfunction associated fatty liver disease (MAFLD) [3]. These chronic liver conditions are characterized not only by sustained hepatic inflammation but also by profound systemic immune dysfunction [4], which plays a pivotal role in shaping the tumor microenvironment and influences the efficacy of both conventional and novel therapeutics.

The pathogenesis of HCC is multifactorial and involves intricate interactions between hepatocytes, stromal elements, immune cells, and microbial signals derived from the gut [5]. However, the precise mechanistic basis of these interactions remains poorly understood, partly due to the scarcity of experimental systems that can recapitulate the complexity of this network.

HCC is often diagnosed radiologically without histological confirmation [6], which limits access to tumor tissue for detailed molecular or immunological analysis. As a result, there is a lack of widely validated biomarkers that reliably predict prognosis or therapeutic response [7]. This knowledge gap is particularly significant in the context of precision oncology, where treatment decisions are increasingly guided by molecular and immune profiling in other malignancies.

Pharmacological management of HCC has evolved substantially over the last decade. Tyrosine kinase inhibitors (TKIs) such as sorafenib and lenvatinib, previously standard first-line agents, have been largely supplanted by immune checkpoint inhibitors (ICIs), which confer significant survival benefits [8]. Combinatorial regimens, including atezolizumab plus bevacizumab and durvalumab plus tremelimumab, are now established first-line options [9]. However, patient responses vary considerably, likely reflecting differences in immune dysregulation, microbial composition, and tumor-intrinsic pathways.

Patient-derived organoids (PDOs) have emerged as a promising platform for precision oncology. PDOs are three-dimensional cultures derived from primary patient tissue that self-organize into structures mimicking the architecture and function of the original tumor [10]. Unlike conventional two-dimensional cultures or immortalized cell lines, PDOs maintain the genetic, transcriptomic, and phenotypic characteristics of the parental tumor [11]. PDOs have been successfully applied to various cancer types to identify actionable mutations, assess drug sensitivity, and investigate mechanisms of therapeutic resistance [12,13].

In HCC, PDOs derived from both surgical specimens and biopsies have demonstrated retention of tumor heterogeneity [14,15].

In this study, we report preliminary data on the development of a PDO model from HCC tissue. In particular, their establishment rate in HCC remains low, with high inter-sample variability and limited data on reproducibility, and little is known about the histopathological and immunohistochemical concordance with the original tumor tissue. Therefore, the aim of this study was to establish a standardized protocol for generating HCC PDOs from resected specimens and to assess their morphological and immunophenotypic fidelity to the primary tumor. We provide a detailed overview of the methodological steps and report preliminary data on PDO yield and histopathological correspondence, which may serve as a reference for future studies in the field.

## 2. Materials and Methods

This was a dual-center, prospective interventional study aimed at generating preliminary data on the immune–microbiota–tumor axis in HCC using PDOs obtained from surgical specimens. Patient recruitment involved the Hepatology Outpatient Clinic of the Digestive System Diseases Center (CEMAD) and the Hepatobiliary Surgery Unit, at the Fondazione Policlinico Universitario Agostino Gemelli IRCCS of Rome, and the Department for the Treatment and Study of Abdominal Diseases and Abdominal Transplants at IRCCS ISMETT of Palermo.

The study received approval from the local Ethics Committee ID 6845 (NCT06929845) and was designed to span a total duration of 24 months.

Eligible participants were adult patients (≥18 years) with a radiologically suspected or histologically confirmed HCC and clinical indication for surgical resection. Written informed consent was obtained from all participants prior to enrollment. Exclusion criteria included age < 18 years, contraindications to hepatic resection, active viral infections, and refusal to provide informed consent.

At screening, patients underwent a comprehensive clinical evaluation, including medical history, physical examination, and review of previous diagnostic reports to assess inclusion and exclusion criteria. Those deemed eligible were enrolled and provided with informed consent. Collected demographic and clinical parameters included: age, sex, weight, height, liver function tests (AST, ALT, γ-GT, ALP, bilirubin), albumin, coagulation profile, complete blood count, and alpha-fetoprotein levels. Additional data comprised etiology of liver disease, fibrosis stage (cirrhosis, advanced fibrosis, steatosis, or healthy liver), alcohol consumption, comorbidities, and ongoing pharmacological treatments. Tumor-related parameters included the date of diagnosis, macroscopic features (e.g., size, number of nodules, macrovascular invasion, metastases), and histological characteristics such as growth pattern, grading, microvascular invasion, capsular invasion, satellite nodules, and Ki-67 expression as a proliferation index. Longitudinal data regarding disease progression, survival, and clinical events were collected from the time of enrollment.

### 2.1. PDOs Culture

After surgical resection, the liver specimens were immediately transferred from the operating room to the pathology unit. Dedicated pathologists assessed the biopsies upon arrival, evaluated tumor quality (as detailed in Section 3.3), and selected the most appropriate tumor fragments for the establishment of hepatocellular carcinoma (HCC) organoids. Each specimen was placed in a sterile dish on ice and carefully inspected. Using sterile scalpels, the pathologists trimmed the tissues to remove macroscopically normal liver parenchyma and any regions not required for organoid culture. Only the visually confirmed tumor lesions were retained for downstream applications. The remaining portions of the biopsies were fixed in formalin at the time of collection for immunohistochemical and histopathological analyses. In parallel, additional portions of tumor tissue were snap-frozen at −80 °C for potential future DNA and RNA extraction. Once the histopathological nature of the tumors was defined by the pathology unit, the corresponding organoids were classified accordingly. RNA-based analyses were not performed in our workflow; therefore, organoid classification relied exclusively on the histopathological features of the matched tumors.

The tumor biopsies selected for organoid culture were processed as quickly as possible, typically within 1 h of surgical resection. To preserve tissue viability, the samples were never allowed to dry out; instead, they were immediately transferred and transported in AdDF+++ culture medium (Advanced DMEM/F12 supplemented with 1× GlutaMAX, 10 mM HEPES, penicillin, and streptomycin) from the pathology unit to the laboratory. Upon arrival, tissue processing began immediately. Liver tumor samples were kept on ice and rapidly washed with AdDF+++ medium. The tissues were mechanically fragmented and subjected to enzymatic digestion at 37 °C using 2.5 mg/mL collagenase IV (Sigma, Darmstadt, Germany) and 0.1 mg/mL DNase I (Sigma) for up to 1 h with orbital shaking at 150 rpm. Digestion was stopped by dilution with cold culture medium, and the resulting material—composed of small cell aggregates—was passed through 70-µm filters to remove debris and undigested tissue fragments. The filtrates were centrifuged at 300× *g* for 5 min in pre-cooled centrifuges at 4 °C. When blood contamination was observed, samples were treated with Red Blood Cell Lysis Solution for 10 min, followed by an additional centrifugation at 300× *g* for 5 min. The interval between tissue digestion and organoid culture initiation was minimized by initiating culture immediately after enzymatic and mechanical dissociation

**Figure 1:**
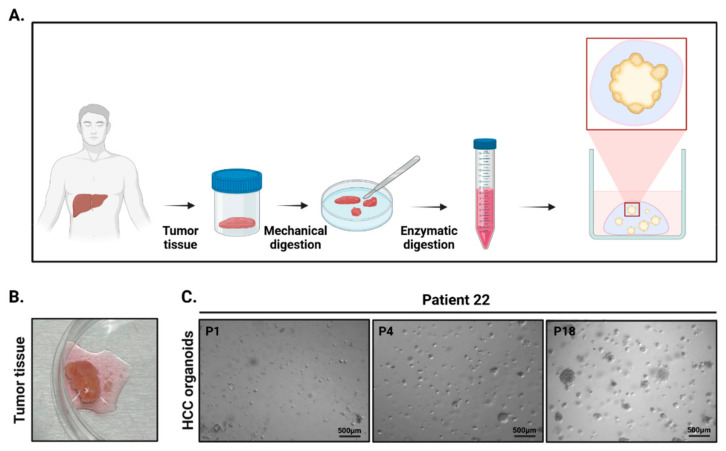
Isolation and culture of organoids (PDOs) derived from human hepatocellular carcinoma (HCC) tissue. (**A**) Schematic representation of the protocol for generating HCC PDOs: tumor tissue is obtained from a patient and subjected to mechanical digestion followed by enzymatic digestion to obtain a cell suspension. The cells are then cultured in a three-dimensional matrix to promote organoid formation. (**B**) Representative image of freshly isolated tumor tissue. (**C**) Microscopic images of organoid formation and expansion at different passages: P1 (passage 1), P4 (passage 4), and P18 (passage 18). Scale bars represent 500 µm. Created in BioRender. Zito, G. (2026) https://BioRender.com/c7s2949 (accessed on 24 December 2025).

The resulting cell aggregates were embedded in pre-cooled Cultrex Basement Membrane Extract (BME), Type 2, Path Clear (Bio-Techne/R&D Systems, Minneapolis, MN, USA; Cat. No. 3532-010-02). This preparation, derived from Engelbreth–Holm–Swarm (EHS) mouse sarcoma, contained laminin, collagen IV, entactin/nidogen, and heparan sulfate proteoglycans at a total protein concentration of 8–12 mg/mL in DMEM without phenol red supplemented with 10 µg/mL gentamicin. The BME remained liquid on ice and polymerized at 37 °C within 20–30 min, enabling stable dome formation for organoid culture. Cell aggregates were seeded in 40 µL drops onto 24-well plates pre-warmed to 37 °C. After BME polymerization, hepatocellular carcinoma organoids were cultured in AdDF+++ medium, consisting of Advanced DMEM/F12 (Thermo Fisher Scientific, Waltham, MA, USA; Cat. No. 12634010), 1× GlutaMAX™ (Cat. No. 35050-061; Gibco, Thermo Fisher Scientific, Waltham, MA, USA), 10 mM HEPES (Cat. No. 15630-080; Gibco, Thermo Fisher Scientific, Waltham, MA, USA), and penicillin–streptomycin (100 U/mL–100 µg/mL) (Cat. No. 15140-122; Gibco, Thermo Fisher Scientific, Waltham, MA, USA). This base medium was supplemented with B-27 (1:50, Thermo Fisher; Cat. No 17504-044), N-2 (1:100, Thermo Fisher; Cat.no 17502-048), nicotinamide (10 mM, Sigma; Cat. No 1003064837), N-acetyl-L-cysteine (1.25 mM, Sigma; Cat. No 616-91-1), [Leu15]-gastrin (10 nM, Sigma; Cat. No 1003377), forskolin (10 µM, Bio-Techne, Minneapolis, MN, USA, Cat. No 66575-29-9), A83-01 (5 µM, Bio-Techne; Cat. No 909910-43-6), Y-27632 (10 µM, Bio-Techne, Cat. No 129830-38-2), EGF (50 ng/mL, Bio-Techne; Cat. No AF-100-15), FGF10 (100 ng/mL, Bio-Techne; Cat. No 0320162-1), HGF (25 ng/mL, Bio-Techne; Cat. No 100-39H), 10% RSPO1-conditioned medium, and 30% Wnt3a-conditioned medium, both produced in-house from expressing cell lines. After 3 days, the medium was changed to Advanced DMEM/F12 containing B-27, N-2, nicotinamide, N-acetyl-L-cysteine, [Leu15]-gastrin, forskolin, A83-01, EGF, FGF10, HGF, 10% RSPO1-conditioned medium, and Y-27632, and was subsequently replaced every 2–3 days. A summary of PDOs generation process is shown in Figure 1. For long-term storage, the organoids were dissociated, resuspended in FBS + 10% DMSO, transferred to cryovials, frozen at −80 °C overnight, and subsequently stored in liquid nitrogen.

### 2.2. Organoids Passaging

Organoids were passaged every 1–4 weeks by adding 400 μL of Cultrex Organoid Harvesting Solution (Bio-Techne/R&D Systems; Cat. No. 3700-100-1) for 45 min at 4 °C with agitation to dissolve the BME2 matrix. The organoids were then dissociated mechanically by pipetting and, when required, enzymatically using TrypLE Express (Gibco, Grand Island, NY, USA) for 5–10 min at 37 °C. Enzymatic activity was quenched with PBS or basal medium, followed by centrifugation at 300× *g* for 5 min at 4 °C. The resulting cell pellets were resuspended in fresh pre-cooled BME2 and replated at a 1:1 to 1:3 ratio, after which the appropriate culture medium was added. All steps were performed on ice to preserve cell viability.

Histological comparisons were performed on organoids at passages P4–P5, and under our culture conditions the organoids consistently retained their characteristic morphology and marker expression for multiple subsequent passages. This stable phenotype, routinely observed across later passages in our hands, was consistent with previous reports demonstrating the long-term stability of liver cancer organoids [16,17].

### 2.3. Immunohistochemical Analysis

Liver cancer biopsies were fixed in formalin and embedded in paraffin as regular clinical practice. HCC organoids were left in 4% paraformaldehyde (PFA) in Phosphate-Buffered Saline (PBS) for 30 min and then washed with PBS to remove excess PFA and embedded in HystoGel (pre-heated to 65 C° to make it liquid). The organoids in HystoGel are then left to rest for 10 min in the refrigerator, fixed in 10% formalin overnight, and processed through dehydration and embedding as follows:

- 70% ethanol, 10 min
- 95% ethanol, 2 × 10 min
- 100% ethanol, 3 × 10 min
- Xylene, 3 × 10 min
- Liquid paraffin, 2 × 15 min.

Five μm sections of paraffin-embedded tissues and HystoGel-embedded PDOs were stained with hematoxylin and eosin (H&E) and immunohistochemistry staining with appropriate primary monoclonal antibodies using the Ventana UltraView Universal DAB Detection Kit (Roche Diagnostics, Tucson, AZ, USA) on the ULTRA instrument (Ventana) BenchMark. The selected antibodies (Table 1), including markers of hepatocellular differentiation, proliferation, and tumor phenotype, are well established in the diagnostic routine laboratory, signals were clearly visible and captured by the Zeiss AxioPhot Microscope (Carl Zeiss, Oberkochen, Germany). Scoring were performed by expert pathologists.

## 3. Results

We enrolled 56 patients, whose characteristics are shown in Table 2. Our patient cohort included mostly moderately to poorly differentiated tumors and classified as grade 2 (G2) and grade 3 (G3) HCC according to World Health Organization (WHO) [18].

### 3.1. HCC-Derived PDOs Maintain the Histological Features of the Tumor of Origin

**Figure 2:**
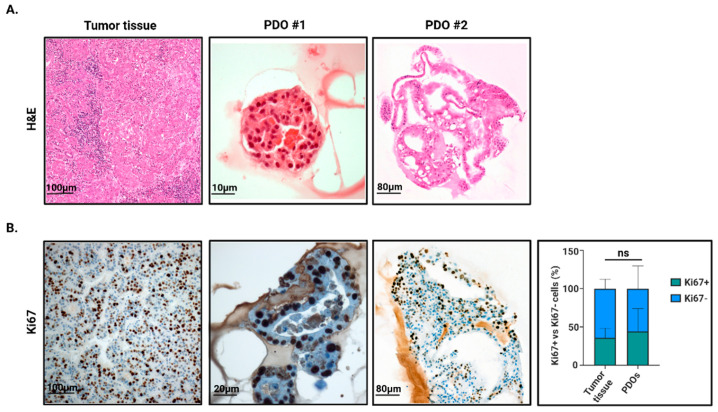
Morphological and proliferative assessment of HCC-derived PDOs. (**A**) Hematoxylin and eosin (H&E) staining. HCC tumor tissue in comparison with HCC-derived PDOs (representative image, scale bar 100 μm for tumor tissue, 10 μm for PDO #1, and 80 μm for PDO #2). (**B**) Immunohistochemical analysis and relative quantification of HCC-derived PDOs expressing the proliferation marker Ki67 (Scale bar 100 μm for tumor tissue, 20 μm for PDO #1, and 80 μm for PDO #2) (n = 8). Created in BioRender. Zito, G. (2026) https://BioRender.com/0vijvow (accessed on 24 December 2025).

While our patient cohort encompassed HCC lesions of all grades, organoid establishment was successful only for G2 and G3 tumors. As PDOs could not be generated from G1 or G4 cases, subsequent characterization and experimental work were necessarily focused on organoids derived from intermediate-grade tumors. Histological analysis of HCC-derived PDOs confirmed the faithful reproduction of the key histopathological features of the tumors of origin. Specifically, the primary tumor shows a disorganized solid/trabecular growth composed of atypical epithelial cells with abundant eosinophilic cytoplasm and enlarged, pleomorphic nuclei. When looking at the corresponding PDOs, a very similar cytological profile is observed: both organoid cultures form cohesive three-dimensional epithelial structures displaying the same enlarged, irregular nuclei and eosinophilic cytoplasm seen in the parental tumor. The architectural disarray that characterizes the primary lesion is largely preserved in vitro, and in PDO#2 this is accompanied by the formation of pseudo-glandular spaces, a feature consistent with the high-grade nature of the original tumor (Figure 2A) [18,19]. Ki-67 staining further supports this morphological continuity. The primary tumor exhibits a high proliferative fraction, with widespread nuclear positivity. Likewise, both PDOs maintain a remarkably elevated proliferative index, with Ki-67-positive cells distributed throughout the organoid structures. Overall, these findings indicate that the PDOs retain key morphological and proliferative features of the tumor they originate from (Figure 2B).

The degree of differentiation is crucial not only for tumor classification and prognosis but also for influencing tumor growth, a key factor in the successful establishment of PDOs.

### 3.2. PDOs Preserve the Immunohistochemical Identity of G2 and G3 HCCs

**Figure 3:**
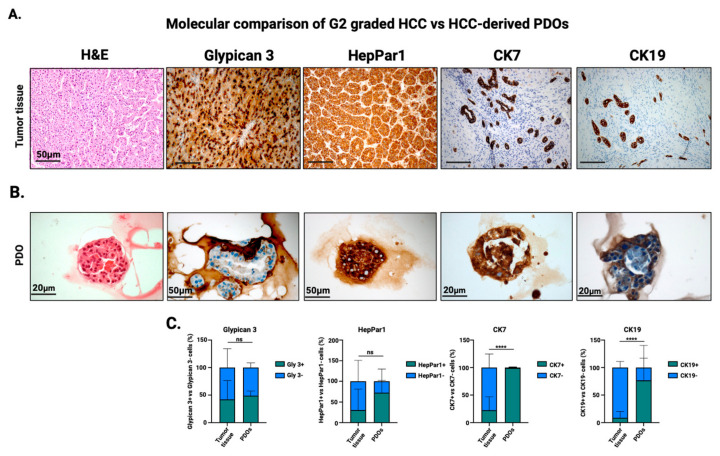
Immunohistochemistry (IHC) analyses of hepatocellular carcinoma (HCC) samples and corresponding patient-derived organoids (PDOs). (**A**) Hematoxylin and eosin (H&E) staining and immunohistochemistry for Glypican-3, HepPar1, CK7, and CK19 in HCC tumor tissue (20× magnification). (**B**) Lower panel: PDOs derived from the same tumor showing comparable morphological and expression patterns (40× magnification). Scale bar 50 μm (tumor tissue and immunostaining of HepPar1 PDO and Glypican3 PDO), 20 μm (H&E PDO, CK7 PDO and CK19 PDO). (**C**) Quantification of the molecular markers evaluated in (**A**,**B**) in both tumor tissues and PDOs (n = 4). Multiple unpaired *t* tests was applied for statistical significance (**** *p* < 0.005; ns = not significant). Created in BioRender. Zito, G. (2026) https://BioRender.com/g650acm (accessed on 24 December 2025).

To assess molecular features of PDOs generated from G2 and G3 HCCs, we conducted immunohistochemical (IHC) profiling of matched PDOs and tumor tissues using clinically validated biomarkers. IHC analysis demonstrated robust expression of Glypican 3 and HepPar1 in the G2 graded primary tumors and PDOs (Figure 3A,B). To note, CK7 and CK19 were found expressed in the tumor, although confined to duct-like structures and not diffusely present among trabecular tumor cells. Interestingly, PDOs express CK7 and CK19, likely reflecting a partial dedifferentiation or lineage plasticity induced by the 3D culture environment. Quantitative analysis of marker expression showed comparable levels of HepPar1 and Glypican-3 in PDOs and primary tissues, indicating that key phenotypic features are well preserved. In contrast, CK19 and CK7 exhibited higher expression levels in PDOs (Figure 3C). These findings confirm that G2-HCC derived PDOs partially recapitulate the molecular and histological phenotype of the original HCC lesion, validating them as a preclinical model for studying tumor biology, drug sensitivity, and personalized therapeutic strategies.

**Figure 4:**
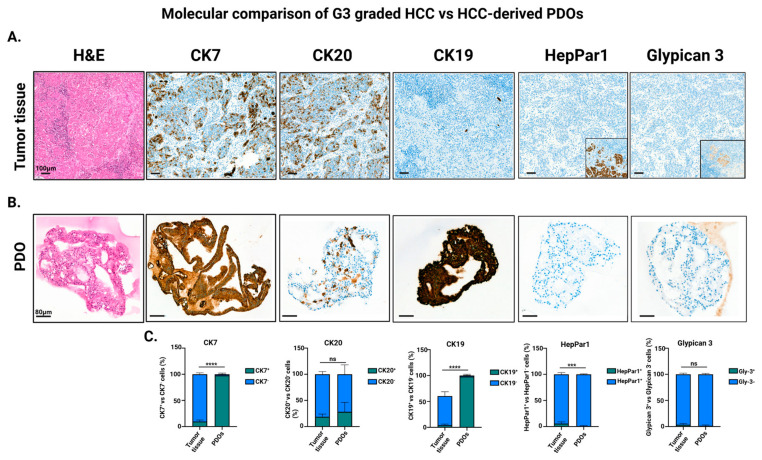
Immuno-histochemical characterization of organoids (PDOs) compared with original tumoral tissue. (**A**) Upper panel: Hematoxylin and eosin (H&E) staining and immunohistochemistry for CK7, CK20, CK19, HepPar-1, and Glypican-3 in the primary HCC tumor tissue (scale bar: 100 µm). For Glypican-3 and HepPar-1, an inset highlights a focal area of positive staining within the tumor, corresponding to the region magnified in the main panel. (**B**) Lower panel: PDOs derived from the same tumor, showing comparable morphological features and marker-expression patterns across the same panel of antibodies (scale bar: 80 µm). (**C**) Quantification of the molecular markers evaluated in (**A**,**B**) in both tumor tissues and PDOs (n = 4). Multiple unpaired *t* tests was applied for statistical significance (*** *p*< 0.001; **** *p* < 0.005; ns = not significant). Created in BioRender. Zito, G. (2026) https://BioRender.com/rzs8w78 (accessed on 24 December 2025).

As reported in the literature, G3 poorly differentiated HCCs are primarily characterized by the loss or focal expression of HepPar1, along with partial or heterogeneous expression of Glypican-3, while they may re-express markers such as CK7 and focal CK20, features typical of certain G3 HCC subclasses with a combined hepatocellular–cholangiocarcinoma phenotype [20,21] (Figure 4A). Consistently, G3-derived PDOs reproduced the immunophenotypic profile of their parental tumors, showing positivity for CK7 and AFP partial CK20 staining, and lack of HepPar1 and Glypican-3 [22,23,24,25] (Figure 4B). Given the focal expression of HepPar1 and Glypican-3 in the tumors (Figure 4A, insets), their absence in PDOs likely reflects sampling from marker-negative regions. Quantitative assessment of marker expression confirmed comparable levels of CK20, HepPar1 and Glypican-3 between PDOs and primary tissues, indicating preservation of key phenotypic features. In contrast, CK19—and, to a lesser extent, CK7—displayed higher expression levels in PDOs (Figure 4C). This increase is consistent with culture-associated dedifferentiation processes that can emerge during in vitro expansion. These observations highlight the capacity of HCC-derived PDOs to retain lineage-specific markers and faithfully reproduce the molecular and histological heterogeneity of HCCs.

### 3.3. Intraoperative Histological Assessment of Biopsy Quality Improves the Success Rate of PDO Generation

To improve the overall PDO establishment rate, we implemented a protocol with a step of rapid intraoperative histological assessment of biopsy quality. This real-time evaluation enabled the selection of highly cellular, viable tumor fragments, which was particularly critical when processing a limited amount of biopsy material. Organoid derivation success increased to 59.4% after the introduction of this, highlighting the value of intraoperative tissue quality control in optimizing PDO workflows for clinical and translational applications.

## 4. Discussion

HCC remains a leading cause of cancer-related mortality globally, with limited treatment options and significant heterogeneity in clinical behavior, molecular profiles, and response to therapies. The advent of precision oncology has underscored the urgent need for models that faithfully recapitulate HCC complexity to enable translational research, drug screening, and biomarker discovery. PDOs have emerged as promising three-dimensional in vitro systems, capable of preserving the histopathological architecture, mutation profile, and intra-tumoral heterogeneity of the tumor of origin [26,27,28]. In various cancer types, including breast, colorectal, pancreatic, and gastric cancers, PDOs have been successfully used to predict treatment responses and explore mechanisms of resistance [29,30,31]. However, in HCC, although promising, the data are still relatively limited and come often from small cohorts or pilot studies [32,33,34,35,36].

In our study, we present preliminary data on the development of a protocol for the generation of HCC-derived organoids from human surgical samples. Our aim was twofold: first, to describe in detail a robust and reproducible methodology tailored for hepatic tissue; and second, to assess the efficiency of organoid establishment, as well as their histological and immunohistochemical fidelity to the original tumor.

The ability to stratify PDOs according to the grade and morphology of the originating tumor confirms the potential of this model to mirror both phenotypic and molecular heterogeneity. This finding is crucial in the context of HCC, a cancer characterized by profound intra-tumoral diversity and typically diagnosed in patients with underlying chronic liver disease [14,15]. The organoids generated using our protocol retained key histological features of the parental tumors, including differentiation pattern and cellular morphology. In several cases, PDOs mirrored the architectural patterns seen in the original tumors, including trabecular, pseudo glandular, or solid growth. This correspondence confirms previous findings and reinforces the role of PDOs as valid preclinical surrogates for HCC modeling [14].

CK7 and CK19 expression in PDOs must be interpreted in the context of the tumor-grade-dependent expression profiles observed in the primary lesions. While CK7/CK19 positivity in G2 tumors was almost exclusively restricted to entrapped biliary ducts, G3 tumors showed CK7 expression in malignant cells with only minimal CK19-positive foci. Accordingly, CK19 expression detected in G3-derived PDOs likely reflects a degree of progenitor-like dedifferentiation occurring in culture. This phenomenon has been previously described in hepatic cell models and likely reflects the influence of the in vitro environment—including growth factors such as epidermal growth factor (EGF), the extracellular matrix, and the mechanical properties of the culture system—on cellular plasticity [37,38,39]. The emergence of CK19 expression in PDOs underscores the need for cautious interpretation of biomarker studies conducted in organoid models and highlights the influence of exogenous signals on cell fate in vitro. These factors represent an intrinsic limitation of PDO-based systems and should be considered when interpreting functional data.

The use of BME as a scaffold, and a defined cocktail of mitogens was instrumental in supporting the growth and propagation of HCC organoids. However, it is important to acknowledge that BME itself, being derived from mouse sarcoma, introduces a non-human matrix component that may influence gene expression and cellular behavior [40]. Future iterations of this model might benefit from the use of fully defined, synthetic matrices to reduce this source of variability [29].

The efficiency of PDO generation from HCC samples is reported to be variable, with success rates ranging from 20% to 50% depending on the source (biopsy vs. surgical), protocol, and tumor characteristics [11,15,41]. In our experience, the success rate of 56.7% was influenced by both tumor grade and tissue handling. Low-grade, well-differentiated tumors exhibited a reduced organoid formation establishment (42.8%) compared to high-grade lesions, which showed a 100% yield of viable PDOs, albeit with greater variability in morphology and growth kinetics. This difference might be attributable to the lower content of stem-like cells or a higher dependency on the hepatic microenvironment observed in G2-grade HCC-derived PDOs. Moreover, we noted that organoid-forming efficiency was often inversely correlated with fibrosis or necrosis in the parental tissue. Samples with extensive stromal reaction or central necrosis tended to yield lower organoid numbers, supporting the need for careful tissue selection and rapid processing to preserve viable tumor cells.

These technical challenges contribute to the low and inconsistent yields reported in the literature and underline the necessity of optimized protocols and trained personnel for successful PDO establishment.

Beyond technical considerations, the biological interpretation of PDO models requires further validation. While PDOs preserve many features of the parental tumor, including morphology and selected immunohistochemical markers, the absence of non-epithelial components—such as immune, endothelial, and stromal cells—limits their ability to recapitulate the complex multicellular interactions within the tumor microenvironment [42]. This is particularly relevant in HCC, where the immune contexture, hepatic stellate cells, and vascular structures all contribute to tumor progression and treatment response [43,44].

Finally, it is important to highlight that most studies to date, including ours, are based on small sample sizes and preliminary datasets. Larger, multi-institutional studies are needed to validate organoid models in HCC and define their predictive and prognostic utility. Moreover, few studies to date have systematically compared the molecular, histological, and immunophenotypic features of PDOs with matched parental tumors, especially using high-resolution technologies such as single-cell RNA sequencing or spatial transcriptomics [15,45,46].

In conclusion, our preliminary results confirm that HCC-derived PDOs can recapitulate key histological and molecular features of the original tumor, including differentiation pattern and marker expression. Although the efficiency of organoid generation remains variable and dependent on tissue quality and tumor characteristics, the model holds promise as a preclinical tool for studying tumor biology and testing therapies. Further work is needed to improve reproducibility, reduce time to culture, and incorporate stromal and immune components to more faithfully reflect the HCC tumor microenvironment. Ultimately, standardized PDO-based platforms could become a valuable addition to the armamentarium of precision oncology for liver cancer.

Moreover, significant limitations remain. First, the time required to establish and expand organoids may preclude their use in time-sensitive clinical decisions, such as first-line therapy selection in advanced HCC. Second, as noted, the absence of the immune and stromal compartments limits mechanistic studies. Third, the use of animal-derived matrices and undefined supplements reduces reproducibility and introduces variability.

## 5. Conclusions

Although PDO technology has shown promising potential in various tumor types, its application in HCC remains technically challenging and variable in terms of success rates and tissue fidelity. In this study, we report preliminary findings from the establishment of HCC-derived organoids, highlighting methodological considerations, histopathological and immunohistochemical concordance with the parental tumor, and overall efficiency. Our data underscore the importance of standardized protocols and systematic validation, especially in the context of a tumor type often diagnosed radiologically without histological confirmation. These insights may contribute to improving the utility of HCC PDOs as translational tools for drug testing, biomarker discovery, and mechanistic studies within the gut–liver–tumor axis framework. Further efforts are warranted to optimize culture conditions and assess their predictive value in clinical settings.

## Figures and Tables

**Table 1:** List of Antibodies used for the organoid characterization.

Marker	Clone	Supplier
CK7	SP52	Roche Diagnostics
CK19	A53-B/A1.26	Cell Marque, Rocklin, CA, USA
CK20	SP33	Roche Diagnostics
HepPar1	121SLE	Cell Marque
Arginase	SP156	Cell Marque
Alpha-fetoprotein	Polyclonal	Cell Marque
Glypican	14 GC33	Roche Diagnostics
Beta-catenin	14	Cell Marque
Ki-67	30-9	Roche Diagnostics

**Table 2:** Clinical characteristics of the HCC patients and establishment of patient-derived organoids (PDOs). Numeric variables are expressed as median and interquartile range, categorical ones as frequency and percentage.

Variable	Value
Patients with HCC	37/56 (66)
Age (yrs)	68 (59.6–74.2)
Sex (F/M)	4 (10.8)/33 (89.2)
Etiology:	
-viral	14 (37.8)
-alcohol	4 (10.8)
-MASLD	15 (40.6)
-other	2 (5.4)
-none	2 (5.4)
Liver disease stage	
-cirrhosis	26 (70.3)
-chronic liver disease	9 (24.3)
-healthy liver	2 (5.4)
Multinodular (>5)	2 (5.4)
Infiltrating pattern	10 (27)
Vascular invasion	
-MVI	10 (27)
-mVI	16 (43.2)
Capsule invasion	14 (37.8)
Microsatellites	7 (18.9)
Grading	
-G1	1 (2.7)
-G2	28 (75.7)
-G3	7 (18.9)
-G4	1 (2.7)
HCC-derived PDOs established	21/37 (56.7)

## Data Availability

The data supporting the findings of this study are available from the corresponding author upon reasonable request, subject to privacy restrictions.

## Abbreviations

The following abbreviations are used in this manuscript:

PDOs	Patient-derived organoids
HCC	Hepatocellular carcinoma
MAFLD	Metabolic-dysfunction associated fatty liver disease
TKIs	Tyrosine kinase inhibitors
ICIs	Immune checkpoint inhibitors
CEMAD	Hepatology Outpatient Clinic of the Digestive System Diseases Center
AdDF+++	Advanced DMEM/F12
BME2	Basement Membrane Extract, Type 2
PFA	Paraformaldehyde
PBS	Phosphate-Buffered Saline
H&E	Hematoxylin and eosin
WHO	World Health Organization
IHC	Immunohistochemical
CK	Cytokeratin
EGF	Epidermal growth factor
G	Grade
RSPO1	R-spondin 1
MASLD	Metabolic dysfunction-Associated Steatotic Liver Disease
